# Toll-Like Receptor and miRNA-let-7e Expression Alter the Inflammatory Response in *Leishmania amazonensis*-Infected Macrophages

**DOI:** 10.3389/fimmu.2018.02792

**Published:** 2018-11-29

**Authors:** Sandra Marcia Muxel, Stephanie Maia Acuña, Juliana Ide Aoki, Ricardo Andrade Zampieri, Lucile Maria Floeter-Winter

**Affiliations:** Departamento de Fisiologia, Instituto de Biociências, Universidade de São Paulo, São Paulo, Brazil

**Keywords:** microRNA, MyD88, NOS2, NO, bone marrow-derived macrophages, post-transcriptional regulation

## Abstract

Parasite recognition by Toll-like receptors (TLRs) contributes to macrophage activation and subsequent control of *Leishmania* infection through the coordinated production of pro-inflammatory and microbicidal effector molecules. The modulation of microRNA (miRNA) expression by *Leishmania* infection potentially mediates the post-transcriptional regulation of the expression of genes involved in leishmanicidal activity. Here, the contribution of TLR signaling to the miRNA profile and gene expression was evaluated in *Leishmania amazonensis*-infected murine macrophages. The infectivity of *L. amazonensis* was higher in murine bone marrow-derived macrophages from mice knockout for myeloid differentiation factor 88 (MyD88^−/−^), TLR2 (TLR2^−/−^), or TLR4 (TLR4^−/−^) than wild type C57BL/6 (WT). *L. amazonensis* infection of WT macrophages modulated the expression of 32% of the miRNAs analyzed, while 50% were upregulated. The absence of MyD88, TLR2, and TLR4 altered the percentage of miRNAs modulated during *L. amazonensis* infection, including the downregulation of let-7e expression. Moreover, the absence of signals mediated by MyD88, TLR2, or TLR4 reduced nitric oxide synthase 2 (*No*s2) mRNA expression during infection. Indeed, the inhibition of let-7e increased levels of the *Nos*2 mRNA and NOS2 (or iNOS) protein and nitric oxide (NO) production in *L. amazonensis*-infected macrophages (4–24 h). The absence of TLR2 and inhibition of let-7e increased the expression of the arginase 1 (*Arg*1) mRNA but did not alter the protein level during infection. However, higher levels of the L-arginine transporters *Cat*2B and *Cat*1 were detected in the absence of Myd88 signaling during infection but were not altered following let-7e inhibition. The inhibition of let-7e impacted the global expression of genes in the TLR pathway by upregulating the expression of recognition and adaptors molecules, such as *Tlr*6, *Tlr*9, *Ly*96, *Tirap, Traf* 6, *Ticam*1, *Tollip, Casp*8, *Map*3*k*1, *Mapk*8, *Nfkbib, Nfkbil*1, *Ppara, Mapk8i*p3, *Hspd*1, and *Ube2*n, as well as immunomodulators, such as *Ptgs*2/*Cox*2, *Csf* 2, *Csf* 3, *Ifnb*1, *Il*6*ra*, and *Ilr*1, impacting NOS2 expression, NO production and parasite infectiveness. In conclusion, *L. amazonensis* infection alters the TLR signaling pathways by modulating the expression of miRNAs in macrophages to subvert the host immune responses.

## Introduction

Protozoan parasites of the genus *Leishmania* are the causative agents of leishmaniases, diseases that affect more than 12 million people worldwide (1). Cutaneous leishmaniasis is widespread in Brazil, the country reporting the greatest number of cases in the Americas, with an estimated incidence of 100,000 cases per year (1). *L. amazonensis* induces cutaneous and/or diffuse cutaneous manifestations (2, 3).

Host-parasite interactions during the innate immune response to *Leishmania* are mediated by the recognition of pathogen-associated molecules (PAMPs) by pattern recognition receptors (PRRs), such as Toll-like receptors (TLRs) that are mainly expressed in phagocytes and antigen-presenting cells, as neutrophils, macrophages and dendritic cells (4, 5). The surface of *Leishmania* spp is covered with molecules that are recognized by TLRs (6), which play central roles in macrophage activation, signaling to induce phagocytosis, parasite healing or persistence and in the control of infections by innate and adaptive immunity (7–11).

The interaction of TLR2 or TLR4 starts the signal transduction cascade through the activation of adaptor molecules, such as MyD88 and Toll-like receptor adapter-inducing interferon β (TRIF/TICAM1) (12), mediating *Leishmania* recognition and modulation of infectivity in macrophages (13–15). The activation of the TLR2 or TLR4 signaling cascade by LPG stimulation and *L. donovani, L. major* or *L. amazonensis* infections modulates the expression of transcription factors, such as nuclear factor kappa B (NF-κB), interferon regulatory factors (IRFs) and mitogen-activated protein kinase (MAPK) that induce the transcription of pro-inflammatory cytokines, such as tumor necrosis factor (TNF), and NO and superoxide production (14, 16–21). However, the mechanism regulating the signal required for the activation of TLR signaling is not completely understood.

The NO produced during the initial steps of the inflammatory response may exhibit leishmanicidal activity (14, 22–24). The outcome of *Leishmania* infection depends on the balance between the induction of NOS2 expression to produce NO and subsequently kill the parasite (25–31) and the induction of ARG1 expression to produce polyamines that promote *Leishmania* survival (28, 32–35). Interestingly, L-arginine is a substrate for both NOS2 and ARG1. Depending on the environmental conditions and immune response, the prevalence of one activity over the other results in the killing of the parasite or its survival. The expression of cationic amino acid transporters (CATs) and their roles in L-arginine uptake into macrophages determines the fate of the infectiveness (36–40).

The immune response is subverted by post-transcriptional regulation mediated by microRNAs (miRNAs), leading to the survival of *Leishmania* (31, 41, 42). The miRNAs are evolutionary conserved small (18–25 nucleotides) non-coding RNAs that fine-tune gene expression by complementary base pairing to the 3′ untranslated region (3′UTR) of a target mRNA, blocking target mRNA translation or inducing its degradation (43–48). The miRNAs are divided into several families, including the *lethal-7* (let-7) family, which was first described in the nematode *Caenorhabditis elegans* as a group of miRNAs responsible for the regulating development in a time-dependent manner (49) and is conserved among various species, including *Drosophila melanogaster* (fruit fly), *Mus musculus* (mouse), and humans (*Homo sapiens*) (50, 51). The let-7e isoform comprises the let-7 family in mice (50–52) and regulates pro- and anti-inflammatory responses during infection or TLR/PAMP stimulation by inducing NF-κB activation and cytokine production (53–57).

In this study, we analyzed the activation of the TLR signaling cascade and the miRNA-mRNA interactions to understand the outcome of *L. amazonensis* infection in C57BL/6 macrophages. TLR2 and TLR4 mediated *L. amazonensis* recognition and infectivity resistance in macrophages. Other MyD88-dependent receptors may participate in macrophage activation in response to *L. amazonensis. L. amazonensis* infection altered the expression of genes involved in TLR signaling, transcription factors and pro-inflammatory molecules. MyD88, TLR2, and TLR4 regulated miRNA expression, such as let-7e, during the course of infection. In addition, the functional inhibition of let-7e altered the activation of the TLR pathway, *Nos2*/NOS2 expression and NO production, impacting the infectiveness of the parasite.

## Materials and methods

### Ethics statement

The protocol used for the animal experiments was approved by the Comissão de Ética no Uso de Animais (CEUA) from Instituto de Biociências of the Universidade de São Paulo (the approval number CEUA-IB: 233/2014). This study was performed in strict accordance with the recommendations in the Guide and Policies for the Care and Use of Laboratory Animals of the São Paulo State (Lei Estadual 11.977, de 25/08/2005) and Brazilian government (Lei Federal 11.794, de 08/10/2008).

### Parasite culture

*L. amazonensis* (MHOM/BR/1973/M2269) promastigotes were maintained in culture in M199 medium (Gibco™, Grand Island, NY, USA) supplemented with 10% heat-inactivated fetal bovine serum (Gibco™), 5 ppm hemin, 100 μM adenine (Gibco™), 100 U penicillin, 100 μg/mL streptomycin (Gibco™), 40 mM HEPES-NaOH (Promega, Madison, WI, USA) and 12 mM NaHCO_3_ (Sigma-Aldrich, St Louis, MO, USA), pH 7.0, for 1 week at 25°C at a low passage (P1 to P5).

### *in vitro* macrophage infections

All experiments were performed using female (6–8 weeks old) C57BL/6 wild type (WT), MyD88^−/−^, TLR2^−/−^, or TLR4^−/−^ mice from Biotério do Departamento de Imunologia, Instituto de Ciências Biomédicas—Universidade de São Paulo and maintained in the Biotério do Departamento de Fisiologia, Instituto de Biociências—Universidade de São Paulo. The animals were sacrificed in a CO_2_ chamber and femurs were collected to obtain bone marrow-derived macrophages (BMDMs). The femurs were washed with 2 mL of cold PBS, and the cells were collected by centrifugation at 1,500 × g for 10 min at 4°C and resuspended in RPMI 1640 medium (LGC Biotecnologia, São Paulo, SP, Brazil), supplemented with penicillin (100 U/mL (Gibco™), streptomycin (100 μg/mL) (Gibco™), 5% heat-inactivated FBS (Gibco™) and 20% L9-29 supernatant. The cells were cultivated for 7–8 days at 34°C in 5% CO_2_ atmosphere. After differentiation, cellular viability was evaluated using flow cytometry (FlowSight® Amnis, MerckMillipore, Darmstadt, Germany), as determined by the presence of 95% F4/80- and CD11b-positive cells.

Approximately 2 × 10^5^ BMDMs were plated into 8-well glass chamber slides (Lab-Teck Chamber Slide; Nunc, Naperville, IL, USA) and incubated at 34°C in 5% CO_2_ atmosphere for the infectivity analysis. Approximately 3 × 10^6^ BMDMs were plated into 6-well plates (SPL, Lifescience, Pocheon, Korea) and incubated at 34°C in 5% CO_2_ atmosphere for analyses of miRNA and mRNA expression. After 18 h of incubation, non-adherent cells were removed by washes with PBS and the infection was performed with promastigotes in the stationary growth phase (MOI 5:1). After 4 h of incubation, non-phagocytosed promastigotes were removed by washes with fresh medium. Thereafter, the infection course was followed for 4, 24 and 48 h. Non-infected macrophages maintained in culture under the same conditions were used as controls. The infectivity was microscopically analyzed after cells were fixed with acetone/methanol (1:1, v:v, Merck, Darmstadt, Germany) for 20 min at −20°C, followed by washes with PBS and staining with DAPI (Invitrogen). The infectivity indexes (rate of infected macrophages multiplied by the average number of amastigotes per infected macrophage) were calculated by randomly counting at least 600 macrophages per slide. For the analysis of RNA expression, cells were washed with PBS, resuspended in QIAzol (Qiagen, Germantown, MD, USA) and stored at −20°C until use.

### RNA extraction, reverse transcription, and miRNA PCR array

RNA was extracted using the miRneasy® Mini kit (Qiagen, Hilden, Germany), according to the manufacturer's instructions. The cDNA templates were produced from mature miRNAs using the miScript II RT kit (Qiagen, Hilden, Germany), according to the manufacturer's instructions. Briefly, 250 ng of total RNA were added to 2 μL of 5X miScript HiSpec Buffer, 1 μL of 10X Nucleic Acids Mix, 1 μL of miScript Reverse Transcriptase Mix and RNAse-free water to a final volume of 10 μL. The mixture was incubated at 37°C for 60 min to insert the poly-A+ sequence at the end of the miRNA sequence (downstream) and anneal a T-tail-tag for the elongation of the cDNA. The enzyme was inactivated at 95°C for 5 min. The reaction was performed in a Thermocycler Mastercycler gradient (Eppendorf, Hamburg, Germany). The cDNAs were stored at −20°C until use.

The miRNA PCR array was performed with 10-fold diluted cDNAs as the template using Mouse Inflammatory Response and Autoimmunity miRNA PCR Array: MIMM-105Z (Qiagen, Germantown, MD, USA) and the miScript SYBR PCR Kit (Qiagen, Hilden, Germany), as previously described (58). Briefly, the pooled mixture was prepared with 1X QuantiTect SYBR Green PCR Master Mix, 1X miScript Universal Primer, 105 μL of cDNA, and RNAse-free water to a final volume of 2,625 μL, and 25 μL was aliquotted into each well. The specific amplification of let-7e-5p by RT-qPCR was performed with 1X QuantiTect SYBR Green PCR Master Mix, 1X miScript Universal Primer, 1X Specific Primer, 5 μL of cDNAs, and RNAse-free water to a final volume of 25 μL. The reactions consisted of the activation of the HotStart Polymerase for 15 s at 95°C and 40 cycles of 15 s at 94°C, followed by 30 s at 55°C and 30 s at 70°C, and were performed in the Thermocycler ABI Prism 7300 instrument (Applied Biosystems, Carlsbad, CA, USA). The relative Ct value was analyzed using the miScript miRNA PCR Array Data Analysis software (www.qiagen.com). Triplicate samples were analyzed for each condition. The average Ct value was calculated to represent the gene expression variation with good accuracy. The geometric average Ct values for the miRNAs were normalized based on the average of SNORD95A. The controls for PCR and RT-qPCR were performed according to the manufacturer's instructions and determined based on efficiency reactions. A negative control containing all reaction components except the reverse transcriptase enzyme was included and subjected to RT-qPCR to confirm the absence of DNA contamination in RNA samples. The Fold Regulation (FR) value is defined as the negative inverse of the fold change [function = −1^*^(1/fold change value)]. FR levels ≥1.2 were considered to indicate upregulation, and levels ≤ 1.2 were considered to indicate downregulation. A Venn diagram was designed Venny 2.1 tool (http://bioinfogp.cnb.csic.es/tools/venny/index.html) (59) and modified to include miRNA or mRNA names inside the areas.

### Reverse transcription and RT-qPCR for analysis of mRNA expression

The cDNA templates for the analysis of mRNA expression were synthesized using 2 μg of total RNA, 20 nmol of random primers (Applied Biosystems, Carlsbad, CA, USA) and water to a final volume of 28 μL. The RNA was incubated at 65°C for 5 min and then cooled to 10°C. Thereafter, mix 2 containing 8 μL of 5X buffer, 2 μL of 10 mM dNTPs and 2 μL of RevertAid™ Reverse Transcriptase (200 U/μL) (Fermentas Life Sciences, Burlington, Ontario, Canada) were added to the reaction, followed by an incubation at 25°C for 5 min and 42°C for 60 min. The enzyme was inactivated at 70°C for 10 min, and the reaction was stored at −20°C until further use. A negative control containing all reaction components except the reverse transcriptase enzyme was included and subjected to RT-qPCR to confirm the absence of DNA contamination in the RNA samples.

For mRNA quantification, we used 100-fold diluted cDNAs as templates. The reaction was performed with 1X SYBR Green PCR Master Mix (Applied Biosystems), 0.4 μM each corresponding primers pair, 5 μL of cDNAs, and RNAse-free water to a final volume of 25 μL. The PCR reaction consisted of 40 cycles of 30 s at 94°C followed by 30 s at 60°C and was performed in the Exicycler™ 96 Real-Time Quantitative Thermal Block (Bioneer, Daejeon, Korea). Triplicate samples were analyzed for each condition. Target gene expression was quantified based on a standard curve prepared from 10-fold serial dilutions of a quantified and linearized plasmid containing the target DNA. The following primers pairs were used for the analysis of mammalian mRNA expression: *Nos2*: 5′-agagccacagtcctctttgc-3′ and 5′-gctcctcttccaaggtgctt-3′; *Arg1:* 5-agcactgaggaaagctggtc-3′ and 5′-cagaccgtgggttcttcaca-3′; *Cat-2b:* 5′-tatgttgtctcggcaggctc-3′ and 5′-gaaaagcaacccatcctccg-3′; *Cat1:* 5′-cgtaatcgccactgtgacct-3′ and 5′-ggctggtaccgtaagaccaa-3′; and *Gapdh*: 5′-ggcaaattcaacggcacagt-3′ and 5′-ccttttggctccacccttca-3′.

### Reverse transcription and TLR PCR array

The cDNA templates were produced from 1 μg of total RNA using RT2 First Strand Kit (Qiagen, Hilden, Germany), according to the manufacturer's instructions. The reactions were performed in a Thermocycler Mastercycler gradient instrument (Eppendorf, Hamburg, Germany) and stored at −20°C until use.

The TLR PCR array was performed with 10-fold diluted cDNAs as templates using RT2 Profiler™ PCR Array Mouse Toll-Like Receptor Signaling Pathway (PAMM-018Z) (Qiagen, Germantown, MD, USA) and RT2 SYBR Green qPCR Mastermix (Qiagen, Hilden, Germany). The reactions were performed with 1X RT2 SYBR Green Master Mix and 100 μL of cDNAs and RNAse-free water to a final volume of 2,700 μL (25 μL/well). The PCR consisted of the activation of the HotStart Polymerase for 10 s at 95°C and 40 cycles of 15 s at 95°C, followed by 1 min at 60°C, and was performed in Thermocycler ABI Prism 7300 (Applied Biosystems, Carlsbad, CA, USA). The relative Ct value was calculated using the RT PCR Array Data Analysis software (www.qiagen.com). Quadruplicate samples were analyzed for each condition. The average Ct value was calculated to represent the gene expression variation with good accuracy. The geometric average Ct values for the mRNAs were normalized based on the average value for the housekeeping gene *B2 microglobulin*. The PCR and RT-PCR controls were performed according to the manufacturer's instructions and determined using efficiency reactions. A negative control containing all reaction components except the reverse transcriptase enzyme was included and subjected to real-time PCR to confirm the absence of DNA contamination in the RNA samples. The Fold Regulation (FR) value is defined as the negative inverse of the fold change [function = −1^*^(1/fold change value)]. FR levels ≥1.2 were considered to indicate upregulation, and levels ≤ −1.2 were considered to indicate downregulation.

### Transfection of miRNA inhibitors

BMDMs were collected, plated and incubated as described in a previously study (58). Thereafter, macrophages were incubated with 100 nM of let-7e-5p inhibitor or the negative control (Ambion, Carlsbad, CA, USA) after a previous incubation with 3 μL of the FugeneHD transfection reagent (Roche, Madison, WI, USA) in 500 μL of RPMI 1640 medium (LGC Biotecnologia, São Paulo, SP, Brazil), supplemented as described above, for 20 min at room temperature. After 24 h of transfection, the cells were infected with *L. amazonensis* promastigotes, as described above.

### Flow cytometry to detect NOS2 and ARG1 expression

Infected BMDMs were fixed with 1% paraformaldehyde (1 h at 4°C) and permeabilized with 0.05% Tween20 for 30 min at 4°C, followed by blocking with Odyssey blocking buffer (LI-COR, Bad Homburg, Germany) for 1 h at room temperature. Then, samples were incubated with 1:200 dilutions of rabbit anti-NOS2 (sc651) or anti-ARG1 (sc20150) antibodies (Santa Cruz, CA, USA) for 16 h at 4°C. Samples were then incubated with 1:300 dilution of FITC-conjugated goat anti-rabbit IgG (F7512, Sigma-Aldrich) antibody for 1 h at room temperature. The fluorescence signal from single cells was measured in channel 2 using FlowSight® Amnis (MerckMillipore, Darmstadt, Germany) and analyzed using the Ideas® Software (Amnis Corporation, Seattle, WA, USA).

### Quantification of NO production

BMDMs were seeded in 24-well plates (SPL) (1 × 10^6^ cells/well) and incubated for 18 h at 34°C in 5% CO_2_ atmosphere. Then, the macrophages were infected for 4 and 24 h, as described above. Cells were detached with 0.5 M EDTA in PBS for 10 min at 37°C, scraped and washed with PBS. Cells were incubated with 5 μM DAF-FM (for NO quantification) (Molecular Probes, Life Technologies, Darmstadt, Germany) in PBS for 30 min at 34°C in 5% CO_2_ atmosphere, as described previously (31, 58). Fluorescence signals from single cells were acquired in channel 2 using FlowSight® Amnis (MerckMillipore, Darmstadt, Germany) and analyzed using the Ideas® Software (Amnis Corporation, Seattle, WA, USA).

### *in silico* analysis

We used the databases miRecords and Dianna tools to determine the interactions between miRNAs and target mRNAs. The miRecords platform (http://c1.accurascience.com/miRecords/) is based on predicted mRNA targets and integrates the predicted targets from various prediction tools: DIANA-microT, MicroInspector, miRanda, MirTarget2, miTarget, NBmiRTar, PicTar, PITA, RNA22, RNAhybrid, and TargetScan/TargertScanS. The Dianna platform (http://diana.imis.athena-innovation.gr/DianaTools/index.php?r=site/index) is based on validated mRNA targets.

### Statistical analysis

Statistical analyses were performed with Student's *t* or ANOVA tests using the GraphPad Prism Software 7 (GraphPad Software, Inc., La Jolla, CA, USA). The obtained *p*-values are indicated throughout the Results section.

## Results

### Absence of the MyD88, TLR2, or TL4 gene increased *L. amazonensis* infectivity

**Figure 1:**
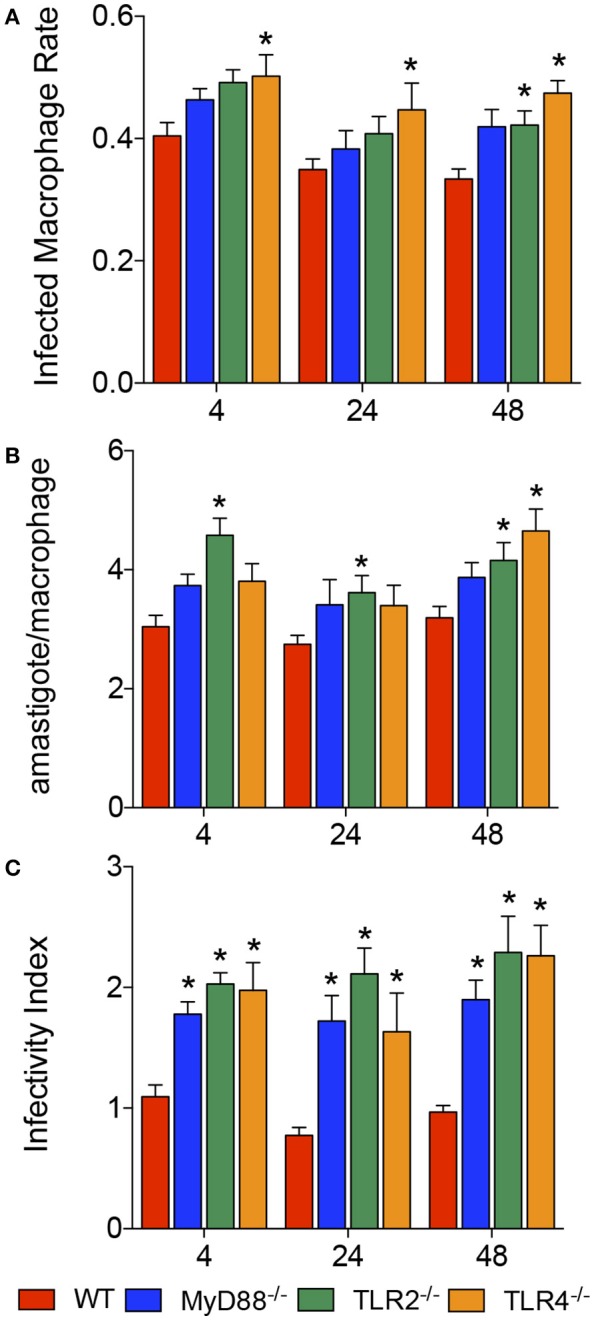
Infectivity of *L. amazonensis* in macrophages from WT, MyD88^−/−^, TLR2^−/−^, and TLR4^−/−^ mice. Results from evaluations of the percentages of infected macrophages **(A)**, numbers of amastigotes per infected macrophage **(B)**, and infectivity index **(C)** in BMDMs from WT (red), MyD88^−/−^ (blue), TLR2^−/−^ (green), and TLR4^−/−^ (orange) mice. Each bar represents the mean ± SEM of the values obtained from three independent experiments (*n* = 6–9). Statistical significance was determined using non-parametric two-way ANOVA. *p < 0.05 compared to WT macrophages.

The roles of MyD88, TLR2 and TLR4 in the recognition of the parasite and defining the fate of *L. amazonensis* infection were evaluated by calculating the percentage of infected macrophages, number of amastigotes per infected macrophage and infectivity index in BMDMs from WT, MyD88^−/−^, TLR2^−/−^, or TLR4^−/−^ mice, after 4, 24, and 48 h of infection (Figure 1). We observed increased numbers of infected macrophages during the course of infection in TLR4^−/−^ macrophages, and an increased rate only after 48 h of infection of TLR2^−/−^ macrophages (Figure 1A). The number of amastigotes per infected macrophage appeared to increase in all knockout mouse strains compared to WT macrophages during the course of infection (Figure 1B). Interestingly, the infectivity index appeared to increase in the MyD88^−/−^, TLR2^−/−^, and TLR4^−/−^ mice during the course of infection compared to infected WT macrophages (Figure 1C), confirming the involvement of TLR2, TLR4, and MyD88 in the recognition of the parasite and defining the fate of *L. amazonensis* infection.

### Modulation of the expression of genes involved in the NO/polyamine pathway depends on TLR signaling

**Figure 2:**
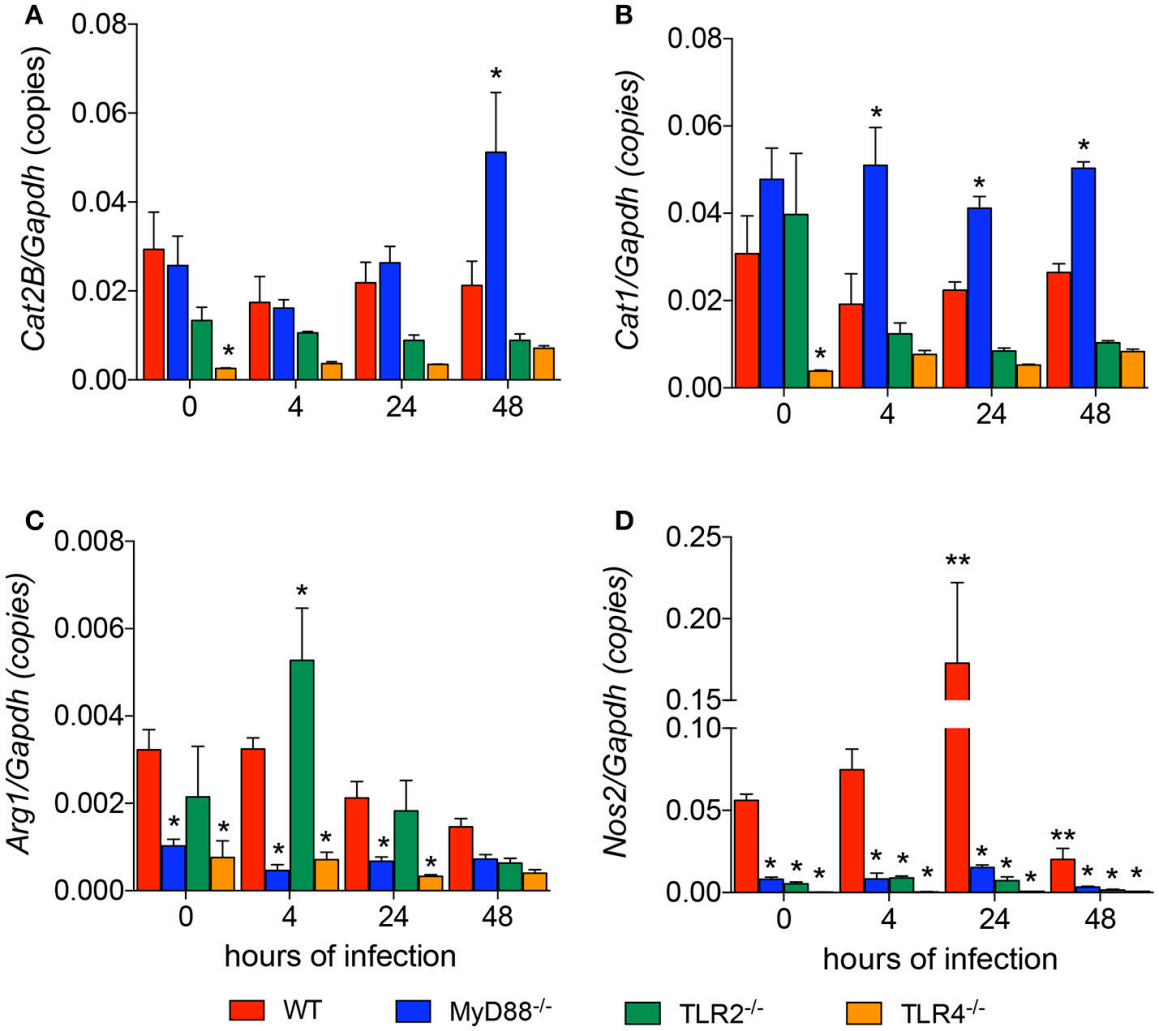
Lack of TLR signaling leads to the differential expression of genes involved in polyamine and NO production. The BMDMs (5 × 10^6^) from WT (red), MyD88^−/−^ (blue), TLR2^−/−^ (green), and TLR4^−/−^ (orange) mice were infected with *L. amazonensis* (MOI 5:1). Four, 24 and 48 h after infection, the copy numbers of the *Cat2B* **(A)**, *Cat1* **(B)**, *Nos2* **(C)**, and *Arg1* **(D)** mRNAs in these macrophages were quantified by RT-qPCR. Uninfected macrophages (0 h) served as the control. Each bar represents the average ± SEM of the values obtained from three independent experiments (*n* = 6–8). Statistical significance was determined using two-tailed Student's *t*-tests. *p < 0.05 all knockout macrophages compared to WT macrophages, **p < 0.05 compared to uninfected macrophages.

We revealed the roles of MyD88, TLR2, and TLR4 in the *L. amazonensis* infection *in vitro*. We next analyzed how TLR signaling interferes with L-arginine metabolism to produce polyamines for parasite replication or to produce NO for parasite killing. First, we analyzed the basal levels of the *Cat1, Cat2B, Arg1*, and *Nos2* mRNAs in each mouse strain and compared the values to the WT strain. TLR4^−/−^ macrophages expressed lower basal levels of the *Cat1, Cat2B, Arg1*, and *Nos2* transcripts (Figures 2A–D). MyD88^−/−^ macrophages showed lower basal levels of the *Arg1* and *Nos2* transcripts (Figures 2C,D). TLR2^−/−^ macrophages did not show differences in the basal levels of any transcript (Figures 2A–D).

We analyzed the levels of those transcripts 4, 24, and 48 h after the *L. amazonensis* infection. The only transcript whose expression was modulated during the course of infection was *Nos2*, which was increased at 24 h and decreased at 48 h after infection (Figure 2D). Compared to WT macrophages, the level of the *Cat2B* transcript was increased in MyD88^−/−^ macrophages 48 h after infection and increased levels of the *Cat1* transcript were observed in MyD88^−/−^ macrophages 4, 24, and 48 h after infection (Figures 2A,B, respectively). In contrast, MyD88^−/−^ macrophages showed decreased levels of *Arg1* and *Nos2* transcripts in all infection periods analyzed (Figures 2C,D, respectively). The comparison between WT and TLR2^−/−^ macrophages showed increased levels of the *Arg1* transcript 4 h after infection and decreased levels of *Nos2* transcript in all infection periods analyzed (Figures 2C,D, respectively). The comparison between WT and TLR4^−/−^ macrophages revealed decreased levels of the *Arg1* and *Nos2* transcripts throughout the course of infection (Figures 2C,D, respectively). Based on these data, MyD88, TLR2, and TLR4 signaling altered the expression of genes involved in polyamine/NO production in *L. amazonensis*-infected macrophages.

### TLR modulates the miRNA profile in *L. amazonensis*-infected macrophages

**Figure 3:**
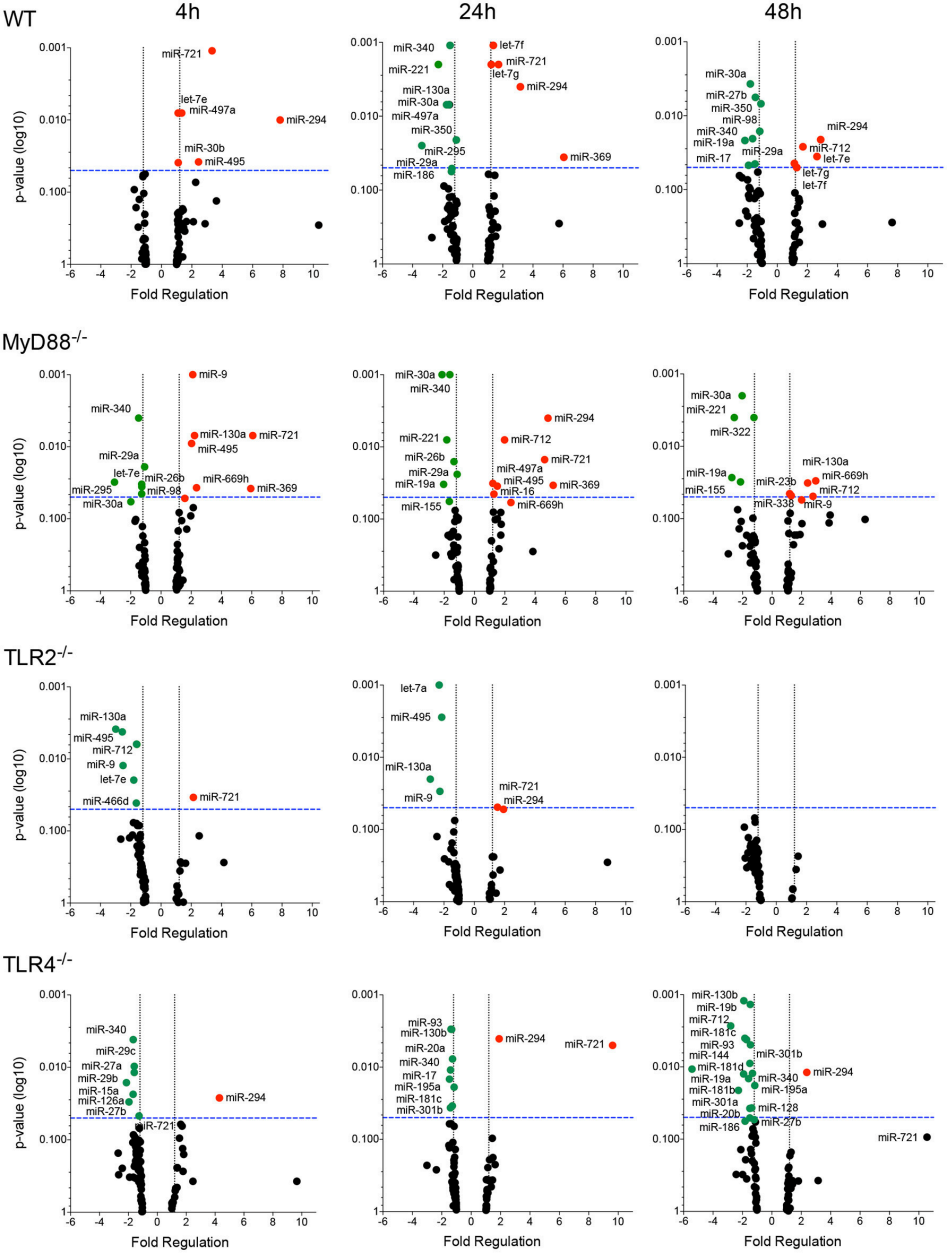
Volcano plot of the miRNA profiles of macrophages from WT, MyD88, TLR2 and TLR4 null mice infected with *L. amazonensis*. Each dot represents the level of one miRNA in BMDMs from WT, MyD88^−/−^, TLR2^−/−^, or TLR4^−/−^ mice infected with *L. amazonensis* for 4, 24, and 48 h. The red dots indicate upregulated miRNAs and green dots indicate downregulated miRNAs. The blue dotted line corresponds to *p* = 0.05, log 10. The relative upregulation and downregulation of miRNAs are presented as boundaries of 1.2 or −1.2 of Fold Regulation, respectively. *P*-values were determined using two-tailed Student's *t*-tests. Representative data from three independent experiments are presented.

**Figure 4:**
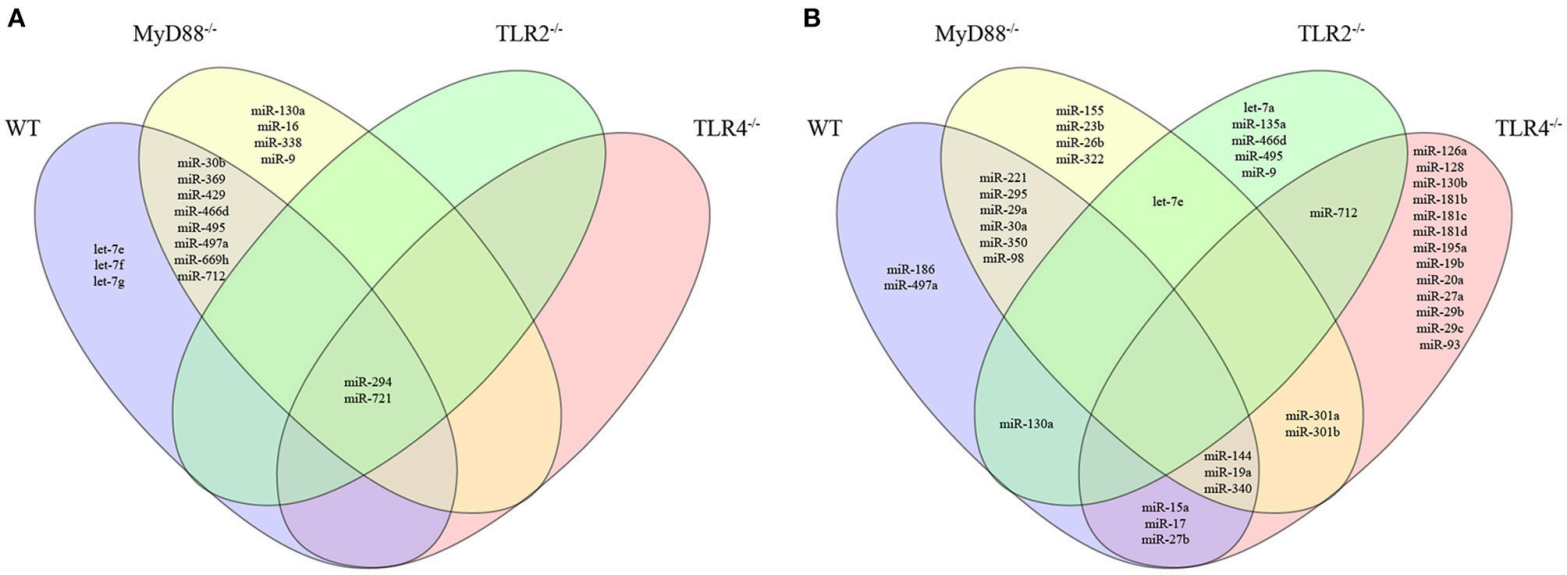
Venn diagram depicting unique and common miRNAs that were significantly differentially expressed in macrophages from WT or MyD88, TLR2 and TLR4 null mice infected with *L. amazonensis*. The miRNA expression levels in infected WT (purple), MyD88^−/−^ (yellow), TLR2^−/−^ (green), or TLR4^−/−^ (red) macrophages were examined. The upregulated **(A)** and downregulated **(B)** miRNAs in each group were compared to uninfected macrophages. The relative up- and downregulation of miRNAs are presented as boundaries of 1.2 or −1.2 of Fold Regulation, respectively. *P*-value < 0.05.

We evaluated the miRNA profile of infected macrophages and the impact of the absence of TLR signaling on miRNA expression by analyzing the miRNA profiles of macrophages from WT, MyD88^−/−^, TLR2^−/−^, or TLR4^−/−^ mice infected with *L. amazonensis* for 4, 24, and 48 h and comparing them to uninfected macrophages (Figures 3, 4, S1; Table S1) to investigate the importance of miRNA-mediated post-transcriptional regulation of gene expression.

The miRNA profile of infected WT macrophages revealed the differential regulation of the expression of 32% (27/84) of the analyzed miRNAs during the course of infection compared to uninfected WT macrophages. Fourteen of the 27 miRNAs were upregulated. Notably, let-7e, miR-182, miR-294, miR-30b, miR-369, miR-429, miR-466d, miR-495, miR-497a, miR-699h, and miR-721 were significantly upregulated 4 h after infection. The upregulation of let-7e, mir-294, and miR-721 was maintained for 24–48 h after infection. The miRNAs let-7f and let-7g appeared to be upregulated 24 and 48 h after infection. Moreover, miR-369 only appeared to be upregulated 24 h after infection, while miR-712 only appeared to be upregulated 48 h after infection (Figure 3; Table S1). Interestingly, we did not detect downregulation of miRNA expression 4 h after infection. On the other hand, 24 and 48 h after infection, we observed downregulation of miR-130a, miR-15a, miR-221, miR-27b, miR-295, miR-30a, miR-340, miR-350 and miR-497a; and miR-17, miR-19a, miR-221, miR-27b, miR-29a, miR-30a, miR-340, miR-350, and miR-98, respectively (Figure 3).

The miRNA profile of infected MyD88^−/−^ macrophages showed the differential regulation of the expression of 35% (30/84) of analyzed miRNAs: 50% of were upregulated and 50% were downregulated (Figure 3). Unexpectedly, the miRNA profile of infected TLR2^−/−^ macrophages revealed low levels of miRNA modulation during the course of infection. Only 12% (10/84) of miRNAs were differentially regulated, and 80% were downregulated. No miRNA modulation was observed 48 h after infection. The miRNA profile of infected TLR4^−/−^ macrophages showed the differential regulation of the expression of 28% (24/84) of the analyzed miRNAs, 92% of these were downregulated (Figure 3).

We constructed a Venn diagram showing exclusively and commonly expressed miRNAs to elucidate the relationships between miRNAs expression among the groups (Figure 4). Notably, miR-294 and miR-721 were both upregulated in all four groups (Figure 4A). In addition, the miRNAs let-7e, let-7f and let-7g were exclusively upregulated in WT cells compared to MyD88^−/−^, TLR2^−/−^, and TLR4^−/−^ cells (Figure 4A). Additionally, let-7e was commonly downregulated miRNA in infected MyD88^−/−^ and TLR2^−/−^ macrophages, and its expression was reduced in TLR4^−/−^ macrophages, but the difference was not significant (*p* = 0.055, Table S1).

The expression of miR-30b, miR-369, miR-429, miR-466d, miR-495, miR-497a, miR-669h, and miR-712 was commonly upregulated in infected WT and MyD88^−/−^ macrophages. Additionally, miR-130a, miR-16, miR-338, and miR-9 were uniquely expressed in infected MyD88^−/−^ macrophages.

Moreover, the Venn diagram showed the following exclusively downregulated miRNAs: miR-182, miR-186 and miR-497a in infected WT macrophages; miR-155, miR-23b, miR-26b, and miR-322 in infected MyD88^−/−^ macrophages; let-7a, miR-135a, miR-466d, miR-495 and miR-9-5p and miR-126a, miR-128, miR-130b, miR-181b, miR-181c, miR-181d, miR-195a, miR-19b, miR-20a, miR-27a, miR-29b, miR-29c, and miR-93-5p in infected TLR4^−/−^ macrophages (Figure 4B). The following miRNAs were commonly downregulated: in WT and MyD88^−/−^ macrophages: miR-221, miR-295, miR-29a, miR-30a, miR-350, and miR-98-5p; in WT and TLR2^−/−^ macrophages: miR-130a; in WT and TLR4^−/−^ macrophages: miR-15a, miR-17, and miR-27b; and in WT, MyD88^−/−^, and TLR4^−/−^ macrophages: miR-144-3p, miR-19a, and miR-340-5p. Notably, miR-301a and miR-301b-3p were commonly downregulated in infected MyD88^−/−^ and TLR4^−/−^ macrophages. Additionally, miR-712 was commonly downregulated in infected TLR2^−/−^ and TLR4^−/−^ macrophages (Figure 4B).

Altogether, our data revealed the differential regulation of the miRNA profiles in *L. amazonensis*-infected macrophages via MyD88, TLR2, and TLR4 signaling.

### *in silico* identification of let-7e target mRNAs

Since we observed the modulation of let-7e expression in the MyD88- and TLR2-deficient mice infected with *L. amazonensis*, we decided to assess the effects of this miRNA on modulating the expression of target mRNAs in the TLR pathway. Based on the results from an *in silico* analysis using miRecords tool, we identified 3,357 predicted interactions for let-7e (Table S2). On the other hand, based on a search using the Dianna tool for experimentally validated targets, let-7e targeted *Tlr*4, *Tbk*1, *Map*2*k*4, *Map*3*k*1, *Chuk, Tnf* , *Tnfpaip*3, *Tnfrsf* 1a, and *Il*10 (60).

### Let-7e affects *L. amazonensis* infectivity by regulating L-arginine metabolism

**Figure 5:**
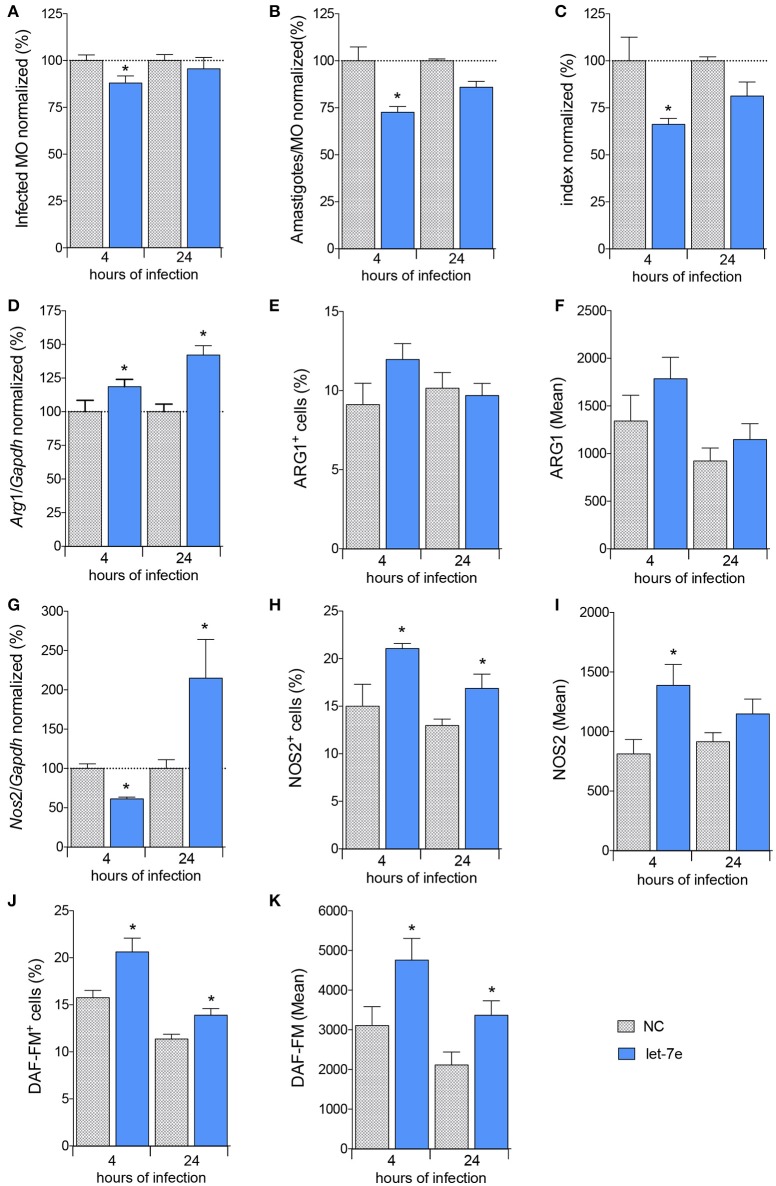
Inhibition of let-7 increases differential gene expression and reduces *L. amazonensis* infectivity. BMDMs were transiently transfected with 100 nM negative control (NC—filed patterned bar) or let-7e-5p inhibitor (let-7e—blue bar). Twenty-four hours after transfection, the cells were co-cultivated with *L. amazonensis* (MOI 5:1) for 4 h, and the cultures were then washed. Four and 24 h after infection, the samples were analyzed for infectivity under the microscope by counting the numbers of infected macrophages **(A)**, amastigotes per infected macrophage **(B)**, and infectivity index **(C)** (*n* = 1,000 macrophages/treatment). RT-qPCR was used to determine the total levels of the *Arg1* **(D)** and *Nos2* **(G)** mRNAs. The percentage of cells expressing ARG1 **(E)** or NOS2 **(H)** and fluorescence intensity of ARG1 **(F)** or NOS2 **(I)** staining were determined using flow cytometry. The percentage of NO-producing cells (DAF-FM^+^ cells—**K**) and fluorescence intensity of NO production per cell (DAF-FM mean—**J**) were also determined using flow cytometry. The values were normalized to the average values of NC-transfected and infected macrophages (100%). Each bar represents the average ± SEM of the values obtained from three independent experiments (*n* = 4–8). Statistical significance was determined using two-tailed Student's *t*-test. *p < 0.05 compared to NC-transfected, infected macrophages.

Next, we analyzed the impact of let-7e expression on *L. amazonensis*-infected macrophages. First, macrophages were transfected with 100 nM let-7e inhibitor or negative-control (NC). Then, macrophages were infected with *L. amazonensis* for 4 and 24 h. Expression assays were used to validate let-7e inhibition, and the inhibition of this miRNA decreased the expression of the miRNA let-7e 4 and 24 h after infection compared to negative control-infected macrophages (Figure S2). The impact of let-7e inhibition on *L. amazonensis* infectivity was also evaluated. As shown in Figures 5A–C, the percentage of infected macrophages, number of amastigotes per infected macrophage and infectivity index at 4 h of infection were reduced by ~10, 30, and 35%, respectively, compared to NC (Figures 5A–C).

We measured the levels of the *Cat2B, Cat1, Arg1*, and *Nos2* transcripts to determine whether let-7e and the subsequent decrease in infectivity required L-arginine metabolism (Figures 5, S2). We did not observe modulation of the levels of the *Cat2* and *Cat1* transcripts among the samples (Figure S2). The level of the *Arg*1 transcript was increased in cells transfected with the let-7e inhibitor 24 h after infection (35%, Figure 5D), but the percentage of cells expressing ARG1 (Figure 5E) and mean intensity of ARG1 (Figure 5F) were not noticeably modified. The level of the *Nos2* transcript was reduced following let-7e inhibition 4 h after infection, but *Nos2* levels were increased 24 h after infection (Figure 5G). Moreover, the percentage of cells expressing NOS2 (Figure 5H), mean intensity of NOS2 (Figure 5I) and NO production were increased in let-7e inhibitor-transfected infected macrophages compared to NC-transfected macrophages, as observed by the percentage of DAF-FM+ cells (Figure 5J) and mean intensity of the DAF-FM signal (Figure 5K). Thus, let-7e inhibition indirectly impacted the expression of genes involved in L-arginine metabolism, promoting NO production and subsequent infectiveness.

### Let-7e impacts global variations in the expression of TLR pathway genes in *L. amazonensis*-infected macrophages

**Figure 6:**
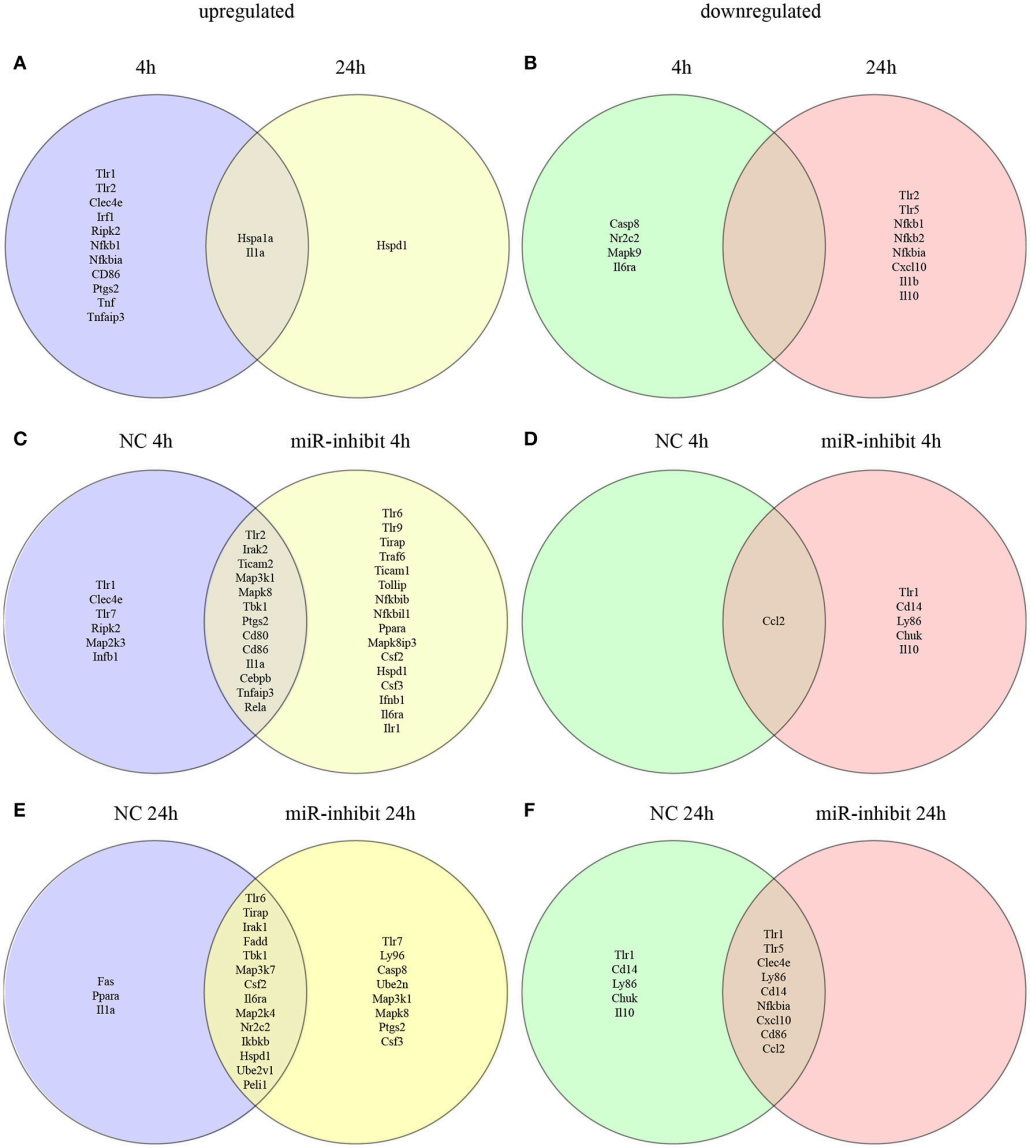
Venn diagram depicting unique and common mRNAs in the TLR pathway and cytokines that were significantly differentially expressed in response to let-7e inhibition in *L. amazonensis*-infected macrophages. BMDMs were transiently transfected with 100 nM negative control (NC) or let-7e-5p inhibitor (let-7e). Twenty-four hours after transfection, cells were co-cultivated with *L. amazonensis* (MOI 5:1) for 4 h, and the cultures were then washed. After 4 and 24 h of infection, cells were analyzed for the expression of mRNAs in the TLR signaling cascade, using RT-qPCR. The upregulated **(A)** and downregulated **(B)** mRNAs were compared at 4 and 24 h after infection. Comparison of mRNA expression in NC- and let-7e inhibitor-transfected cells: upregulated at 4 h **(C)** or 24 h **(D)** or downregulated at 4 h **(E)** or 24 h **(F)**. The relative up- and downregulation of miRNAs are presented as boundaries of 1.2 or −1.2 of Fold Regulation, respectively. *P*-value < 0.05.

We evaluated and compared the transcript levels of molecules involved in recognition/binding and downstream signaling, such as adapters, transcription factors, cytokines/chemokines and cytokine receptors, in infected WT macrophages or negative control (NC)- and let-7e-inhibited macrophages 4 and 24 h after *L. amazonensis* infection and compared the results to uninfected macrophages to determine the role of let-7e in regulating the expression of molecules in the TLR pathway involved in pathogen recognition and the establishment of an infection (Figures 6, S3; Table S3).

First, we compared the expression of genes in the TLR signaling cascade in infected macrophages 4 and 24 h after infection (Figures 6A,B, S3). A substantial number of genes were upregulated 4 h after infection: *Tlr*1, *Tlr*2, C-type lectin domain family 4-member e (*Clec*4e/*Mincle*), heat shock protein 1A (*Hspa*1a/HSP70), interferon regulatory factor 1 (*Irf* 1), receptor-interacting protein kinase 2 (*Ripk*2), nuclear factor kappa B subunit 1 (*Nfkb*1), NF-kB inhibitor alpha (*Nfkbia*/*Ikbalpha*), CD86 antigen (*Cd*86), interleukin 1 alpha (*Il1*a), prostaglandin-endoperoxide synthase 2 (*Ptgs*2/*Cox*-2), tumor necrosis factor (*Tnf* ), and TNF alpha-induced protein 3 (*Tnfaip*3). Of these genes, only the upregulation of *Hspa*1a and *Il*1a was maintained 24 h after infection, and the expression of the heat shock protein 1 (*Hspd*1) gene was upregulated (Figure 6A).

A few genes were downregulated 4 h after infection: caspase 8 (*Casp*8), nuclear receptor subfamily 2, group C, member 2 (*Nr*2*c*2/TAK1), mitogen-activated protein kinase 9 (*Mapk*9/JNK2) and interleukin 6 receptor-alpha (Il6ra). The following genes were downregulated after 24 h: *Tlr*2, *Tlr*5, *Nfkb*1 (p105/p50), *Nfkb*2 (p100/p52), Nfkbia/Ikbalpha, chemokine CXC-motif ligand 10 (Cxcl10), interleukin 1 beta (*Il*1*b*), and interleukin 10 (*Il*10) (Figures 6B, S3; Table S3).

In the infected macrophages transfected with the let-7e inhibitor we detected the following upregulated genes 4 h after infection compared to infected NC-transfected macrophages: *Tlr*6, *Tlr*9, Toll-interleukin 1 receptor (TIR) domain-containing adaptor protein (*Tirap*), Tnf receptor-associated factor 6 (*Traf* 6), Toll-like receptor adaptor molecule 1 (*Ticam*1), *Tollip, Nfkbib*/TRIP9, *Nfkbil*1, peroxisome proliferator activated receptor alpha (Ppara), *Mapk*8*ip*3/*Jip*3, *Ptgs*2/Cox2, colony-stimulating factor 2 (*Csf* 2/GMCSF), Hspd1, Csf3, interferon beta 1 (*Infb*1), *Il*6ra and *Il*r1 (Figure 6C). A few genes were downregulated: *Tlr*1, CD14 antigen (*Cd*14), lymphocyte antigen 86 (*Ly*86/MD-1), conserved helix-loop-helix ubiquitous kinase (*Chuk*/IKBKA), and *Il*10 (Figure 6D).

At 24 h after infection, the following genes were upregulated in infected macrophages transfected with the let-7e inhibitor compared to macrophages transfected with NC: *Tlr*7, *Ly*96/MD-2, *Casp*8, *Map*3*k*1, *Mapk*8, *Ptgs*2/*Cox*2, *Csf* 3/GCSF, and the ubiquitin-conjugating enzyme E2N (*Ube*2n). Indeed, let-7e inhibition did not alter the downregulated genes compared to NC-transfected infected macrophages.

Additionally, let-7e inhibition increased the levels of predicted target mRNAs, including *Traf* 6, *Ppara, Mapk*8*ip*3/*Jip*3, *Map*3*k*1, and *Ube*2*n*, in infected macrophages (Tables S2, S3). Based on these data, the expression of genes involved in TLR pathways is altered during *L. amazonensis* infection. Indeed, let-7e inhibition during *L. amazonensis* infection impacts global variations in the expression of genes TLR pathways (Figure 6) and infectiveness (Figures 5A–C).

## Discussion

The importance of *Leishmania*-TLR interactions in enabling the activation of macrophage during healing and protection against leishmaniases has been extensively studied; however, the findings are very controversial. Macrophages have been reported to recognize the parasite, inducing a pro-inflammatory response and microbicidal mechanisms. TLR4 induces TIRAP-MyD88 signaling and then endocytosis, whereas TRIF-TRAM is activated in early endosomes (12). The trafficking of TLRs 3, 7, 8, and 9 from the endoplasmic reticulum to the endolysosomal compartment collaborates in the recognition of PAMPS and DAMPs (12). Here, we reported the roles of TLR2, TLR4, and the adaptor molecule MyD88 in mediating the phagocytosis of *L. amazonensis* and conferring resistance to infection, in contrast to the higher infectivity index observed in the absence of those molecules.

The recognition of virulence factors, such as LPG and αGalβ1, 4Manα-PO(4)-containing phosphoglycans from *L. major*, by TLR2 requires the adaptor MyD88 (14, 61). Stimulation of TLR2 induces the production of pro-inflammatory cytokines, such as TNF and IL-6, reactive oxygen species (ROS) (61, 62), and suppressors of the cytokine signaling family members SOCS-1 and SOCS-3 (15, 61, 62). P8 proteoglycolipid complex (P8 PGLC) from *L. pifanoi* induces the production of TNF and IL-12 in macrophages via TLR4/MD2-CD14 signaling. Additionally, TNF production and the parasite burden depend on TLR2 and MyD88 (20). Furthermore, MyD88 assists in the recognition of *L. donovani* and maturation of dendritic cells during infection (63). Indeed, TLR2, TLR3, MyD88, and IRAK-1 are involved in the phagocytosis of *L. donovani* and production of TNF-α and NO, although only IFN-γ-primed macrophages present leishmanicidal activity (14).

Several studies have described the participation of transcription factors and miRNAs in the transcriptional and post-transcriptional regulation of gene expression, respectively (64). *L. major* (65), and *L. donovani* (41, 42) have been reported to modulate miRNA expression in infected human macrophages. In BALB/c macrophages infected with *L. amazonensis*, the miRNA profile was altered and the involvement of miR-294, miR-497a and miR-721 in infection was confirmed (31). The results presented in this study corroborate these data, showing that *L. amazonensis* infection regulated the miRNA profiles and let-7e expression in infected C57BL/6 WT, MyD88^−/−^, TLR2^−/−^, and TLR4^−/−^ macrophages. The miRNAs let-7e, let-7f, let-7g miR-30b, miR-369, miR-495, and miR-712 were only upregulated in infected C57BL/6 WT macrophages, while TLR2-, TLR4-, or MyD88-deficient mice exhibited alterations in the expression of other miRNAs during *L. amazonensis* infection. Based on this evidence, the expression of let-7e, let-7f, and let-7g require MyD88, TLR2, and TLR4 signaling during *L. amazonensis* infection, and our findings reveal the role of the TLR pathway in the transcriptional and post-transcriptional regulation of gene expression during *Leishmania* infection.

The miRNA let-7 family is highly conserved from bacteria to mammals and modulates the expression of some anti-inflammatory effector molecules, such as IL-10 (66), and pro-inflammatory effector molecules, such as IL-6 (let-7a) (67) and IL-13 (let-7d) (68). Infections with *Mycobacterium* and *Neisseria* induce let-7e expression in macrophages (56, 57). The expression of let-7e modulates p65 NF-κB activation (54) and phosphoinositide-3 kinase/serine-threonine protein kinase (Pi3k/Akt) (53, 57). Indeed, let-7e downregulated TLR4 and reduced pro-inflammatory signals, such as TNF, IFN-alpha, IL-6, IL-17, MCP-1, MIP-1, and IP-10 (53, 55, 57). Moreover, let-7e targeted the suppressor of cytokine signaling 4 (SOCS4) and regulated IL-13 expression in an allergic process (69).

Here, *L. amazonensis* infection in C57BL/6 WT macrophages increased the levels of recognition molecules, such as *Tlr*1, *Tlr*2, *Tlr*5, and *Clec*4e/*Mincle*, supporting the involvement of TLRs in the identification and phagocytosis of the parasite. Moreover, the increased expression of adaptor and effector molecules, such as *Hspa*1a, *Hspd*1, *Irf* 1, *Ripk*2, *Nfkb*1, *Nfkb*2, *Nfkbia*/*Ikbalpha*, as well as immunomodulators, such as *Cxcl*10, *Cd*86, *Il1*a, *Il1*b, *Tnf* , *Il*10, and *Tnfaip*3, and the decrease in the expression of *Casp*8, *Nr*2*c*2/TAK1, *Mapk*9/JNK2, and Il6ra during infection indicates that *L. amazonensis* modulated macrophage activation.

**Figure 7:**
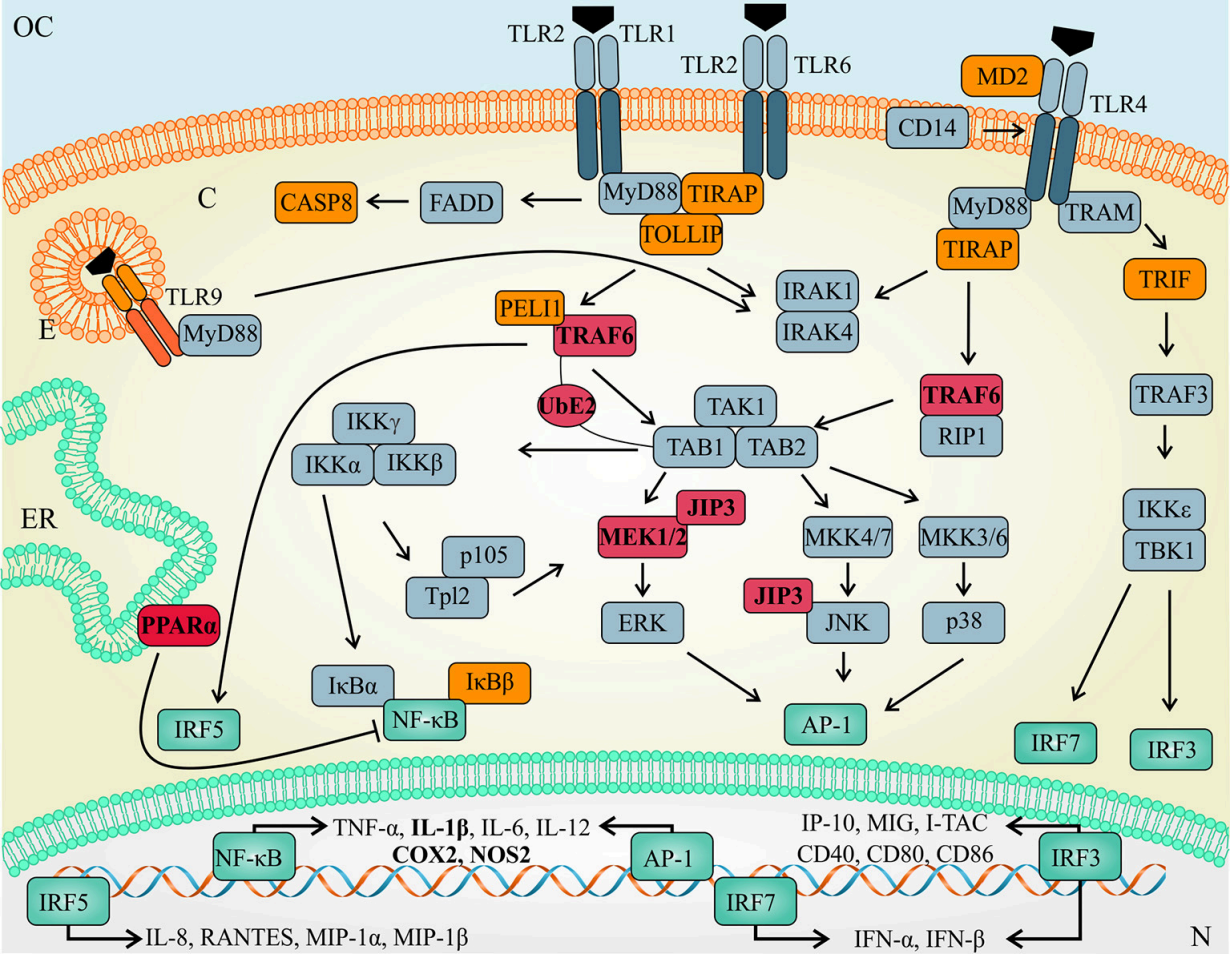
Schematic of the TLR pathway and let-7e target mRNAs. TLR activation is regulated by let-7e-mediated post-transcriptional mechanisms acting directly (predicted targets in red) or indirectly (molecules in orange) on mRNA/protein expression by modulating the expression of molecules involved in recognition, adaptors and effector molecules, as well as transcription factors (green) that regulate the expression of cytokines, chemokines and other immunomodulators.

Interestingly, functional inhibition of let-7e during *L. amazonensis* infection reduced the infectivity and impacted the global expression of mRNAs involved in the TLR signaling cascade. As shown in Figure 7, the levels of predicted targets, such as *Traf* 6, *Ppara, Mapk*8*ip*3/*Jip*3, *Map*3*k*1, and *Ube*2n (represented in red), and the following indirect targets (represented in orange) were increased: recognition molecules: *Tlr*9 and *Ly*96/MD-2; adaptors and effectors: *Tirap, Ticam*1, *Tollip, Casp*8, *Map*3*k*1, *Peli1, Nfkbib*/TRIP9, *Nfkbil*1, and *Hspd*1; and immunomodulators: *Ptgs*2/*Cox*2, *Csf* 2/GMCSF, *Csf* 3/GCSF, and *Ilr*1. Nevertheless, the previously validated let-7e targets *Tnfpaip*3, *Map*2*k*4, *Tbk*1, and *Tnf* were upregulated during *L. amazonensis* infection, whereas the decrease in *Chuk*/IKBKA and *Il*10 expression during infection was not reversed by let-7e inhibition.

The roles of TLR/IL-1R signaling pathways in the ubiquitin-dependent NF-κB activation and subcellular trafficking have been extensively studied. The ubiquitination system is based on the ubiquitin-activating E1 enzyme, ubiquitin-conjugating E2 enzymes, and ubiquitin E3 ligases. The E2 UBE2n dimerizes with UBE2v, forming K63-linked ubiquitin chains to polyubiquitinate adaptor molecules, such as TRAF6, which is recruited to the TAB2/TAK1 complex and activates NF-κB, as shown in Figure 7 (70–73). However, the role of ubiquitination in the immune response to *Leishmania* has not been studied. Another molecule, PPARα was not previously reported to be involved in *Leishmania* infection. However, TLR4 transcription and signaling are negatively regulated via PPARα, which inhibits the NF-κB signaling pathway (74). PPARα also regulates the expression of genes involved in free fatty acid metabolism, could bind to TLR4 and interfere with the inflammatory response. In addition, Tollip is a negative regulator of the TLR signaling pathway, regulates the production of the pro-inflammatory cytokines IL-6 and TNF (75) and potentially influences the response to cutaneous *L. guyanensis* infection (76). Mapk8ip3/JIP3 helps the TLR4 signaling cascade enhance the activation of the JNK and MEK1/2 pathways (77, 78).

Indeed, an early step in the infection of human macrophages with *L. amazonensis* and *L. major* occurs the upregulation of pro-inflammatory cytokine genes, such as *Il*1b, *Tnf* , and *Il*6, as well as other molecules involved in tissue growth and repair, such as *Csf* 2 and *Ptgs*2/*Cox*2 (79). Similarly, *L. major* infection of murine macrophages increases the levels of *Tnf, Il*1, *Il*6, *Nos*2, *I1rap*, and *Csf* 3, as well as molecules in the NF-κB and MAPK signaling pathways (21).

Here, TLR2 and TLR4 induced the expression of the *Cat2B* and *Cat1* mRNAs in *L. amazonensis*-infected macrophages, while a MyD88 deficiency induced their expression, helping control L-arginine uptake. L-arginine uptake mediated by *Cat*2B helps control *L. amazonensis* infectiveness (40). The regulation of polyamine production by *Arg1* mRNA expression depended on TLR4 and MyD88. Thus, *Nos2* expression depends on TLR2, TLR4, and MyD88 molecules during infection and its activation in C57BL/6 mouse macrophages may enhance the resistance to infection, as previously shown in murine macrophages lacking *Nos2* (80). Notably, let-7e inhibition regulated *Arg*1 and *Nos2* expression during infection, corroborating the importance of the TLR signaling cascade in the mechanism regulating *Arg1* and *Nos2* expression at the transcriptional level. The NF-κB pathway regulates *Nos2* expression at the transcriptional level (81). In cells infected with *L. major* and *L. amazonensis* increasing in the nuclear translocation of the NF-κB p50/p50 dimer correlates with the decrease in the levels of the *Nos2* and *Cat2B* transcripts (16). In response to an *L. amazonensis* infection, miR-294-3p and miR-721 post-transcriptionally degrade the *Nos2* mRNA in BALB/c macrophages (31). *Leishmania* also induces L-arginine uptake mediated by amino acid permease 3 (82–84), amino acid metabolism via arginase activity (85, 86), and *NOS-like* activity (87). L-arginine is essential for parasite survival and interferes with macrophage activation during infection (88). Indeed, parasite arginase competes with L-arginine and reduces *Nos2* expression and NO production in *L. amazonensis*-infected hosts (31).

NOS2 activity induces NO production to trigger the macrophage-mediated inflammatory response and kill the parasites (39, 89–91). The balance between polyamine production via ARG1 and NO production via NOS2 influences the fate of infectivity in different mouse models. IL-12 production and NOS2 expression reduce the infectivity in C57BL/6 mice. In contrast, IL-4 production and *Arg1* expression increase the infectivity in BALB/c mice (37, 38, 88). The cytokines IL-12 and IL-4 induce CAT2B expression and the uptake of L-arginine, a substrate of ARG1 and NOS2, altering outcome of infection, while the negative regulation of CAT2B expression also reduces the infectivity of *L. amazonensis* (40).

Interestingly, TLR4 contributes to the control of parasite growth in the initial and later steps of *L. major* infection (13). In addition, *L. major* infection induces NOS2 expression by activating TLR4, although ARG1 expression is independent of TLR4 during infection *in vivo* and *in vitro* (13). TLR9 induces NOS2, IL-12 and IFN-gamma expression and helps control the parasite burden in *L. major*-infected BALB/c and C57BL/6 mice (92). However, MyD88 signaling increases the ratio of IL-12/IL-10 in *L. amazonensis-* and *L. braziliensis*-infected dendritic cells (15). MyD88 mediates the resistance to *L. braziliensis* infection in C57BL/6 mice, but TLR2 enhances regulation of the immune response by reducing the ratio of IL-12/IL-10 (15).

*L. amazonensis* infection has been reported to regulate the activation of MAPK in BALB/c macrophages by regulating IL-10 production (28). However, p38-MAPK activation reduces the infectivity of *L. donovani* (93). On the other hand, *L. mexicana* glycosylphosphatidylinositol-anchored lipophosphoglycan (LPG)/TLR stimulation regulates the activity of the MAPK and NF-κB pathways to alter IL-12 production (94, 95). The chaperone HSP60 serves as danger signal for stressed or damaged cells and its effects are potentially mediated by its anti-apoptotic activity induced by the formation of the HSP60/pC3 complex (96). TNF or TLR signaling leads to the formation of a Fas-associated protein with death domain (FADD)/pro-caspase 8 complex and activation of caspase 8, which induces apoptosis via direct cleavage of downstream caspases, caspase-3/7 (97). In response to an *L. major* infection, the inhibition of caspase 8 increases the production of Th1 and Th2 cytokines and NO (98), in contrast to the antiapoptotic signaling mediated by caspase 8 during *L. infantum* infection (99). The modulation of the expression of mRNAs involved in the TLR pathway impacted the infectivity of *L. amazonensis*.

Our data corroborated the idea that *Leishmania* infection may regulate the TLR pathway and subsequent cytokine and chemokine production, as well as NO/polyamine production to control macrophage activation and infectiveness. We emphasize the importance of TLR signaling and miRNA expression during infection and the integration of these molecules in the mechanism regulating gene expression required to determine the fate *Leishmania* infection, as well as the identification of potentially new targets to control infectivity and pathogenesis.

## Author contributions

SM, JA, and LF-W conceived and designed the experiments. SM, JA, RZ, and SA performed the experiments. SM, JA, and LF-W wrote the original article. SM designed the figures. SM, JA, RZ, SA, and LF-W revised the article.

### Conflict of interest statement

The authors declare that the research was conducted in the absence of any commercial or financial relationships that could be construed as a potential conflict of interest.

## Supplementary material

Figure S1 Scatter plots of the miRNA profiles of C57BL/6 (WT), MyD88^−/−^, TLR2^−/−^, and TLR4^−/−^ murine BMDMs infected with *L. amazonensis*. Each dot represents one miRNA in BMDMs infected with *L. amazonensis* for 4, 24, and 48 h. The red dots indicate upregulated miRNAs and green dots indicate downregulated miRNAs in both comparisons. Blue dots indicate upregulated miRNAs in WT macrophages compared to knockout cells and orange dots indicate downregulated miRNAs in WT macrophages compared to knockout cells. The relative up- and downregulation of miRNAs are reported as boundaries of 1.2 or −1.2 of Fold Regulation, respectively. Only significant (*p* < 0.05) Fold Regulation values are presented.

Figure S2 Inhibition of let-7 functions. BMDMs were transiently transfected with 100 nM negative control (NC) or let-7e-5p inhibitor. Twenty-four hours after transfection incubation, cells were co-cultivated with *L. amazonensis* (MOI 5:1) for 4 h, and the cultures were then washed. After 4 and 24 h of infection, the samples were analyzed for let-7e levels normalized to SNORD95 **(A)**, and levels of the *Cat2B* **(B)**, and *Cat1* **(C)** mRNAs using RT-qPCR. The values were normalized to the average values of NC-transfected, infected macrophages (100%). Each bar represents the average ± SEM of the values obtained from three independent experiments (*n* = 3–6). Statistical significance was determined using two-tailed Student's *t*-tests. ^*^*p* < 0.05 compared to negative control-transfected, infected macrophages.

Figure S3 Expression of mRNAs encoding proteins in the TLR pathway molecules and cytokines in *L. amazonensis*-infected macrophages. The BMDMs (5 × 10^6^) from WT mice were infected with *L. amazonensis* (MOI 5:1). Each dot represents one mRNA in BMDMs from WT mice infected with *L. amazonensis* for 4 and 24 h. Blue dots indicate upregulated mRNAs and orange dots indicate downregulated mRNAs. The blue dotted line corresponds to *p* = 0.05, log 10. The relative up- and downregulation of miRNAs are presented as boundaries of 1.2 or −1.2 of Fold Regulation, respectively. *P*-values were determined using two-tailed Student's *t*-test. Representative data from three independent experiments are shown.

Table S1 C57BL/6 (WT), MyD88^−/−^, TLR2^−/−^, and TLR4^−/−^ murine macrophages during *L. amazonensis* infection. FR (Fold Regulation) represents the fold change results at a biological magnitude. Values >1.2 indicate positive regulation and values < -1.2 indicate negative regulation.

Table S2 Predicted target mRNAs of the let-7e miRNA. The miRNA-mRNA interactions predicted using miRecord tools (http://c1.accurascience.com/miRecords/) were based on the integration of the predicted targets obtained from at least three of the following prediction tools: DIANA-microT, MicroInspector, miRanda, MirTarget2, miTarget, NBmiRTar, PicTar, PITA, RNA22, RNAhybrid, and TargetScan/TargertScanS.

Table S3 Let-7e inhibition alters the levels of mRNAs in the TLR pathway in WT macrophages during *L. amazonensis* infection. FR (Fold Regulation) represents the fold change results at a biological magnitude. Values >1.2 indicates positive regulation and values < -1.2 indicate negative regulation.
