# Supplementary figures and images for: Toll-Like Receptor and miRNA-let-7e Expression Alter the Inflammatory Response in Leishmania amazonensis-Infected Macrophages
Source: Front Immunol. 2018 Nov 29;9:2792. doi: 10.3389/fimmu.2018.02792 (PMC6283264; doi:10.3389/fimmu.2018.02792)

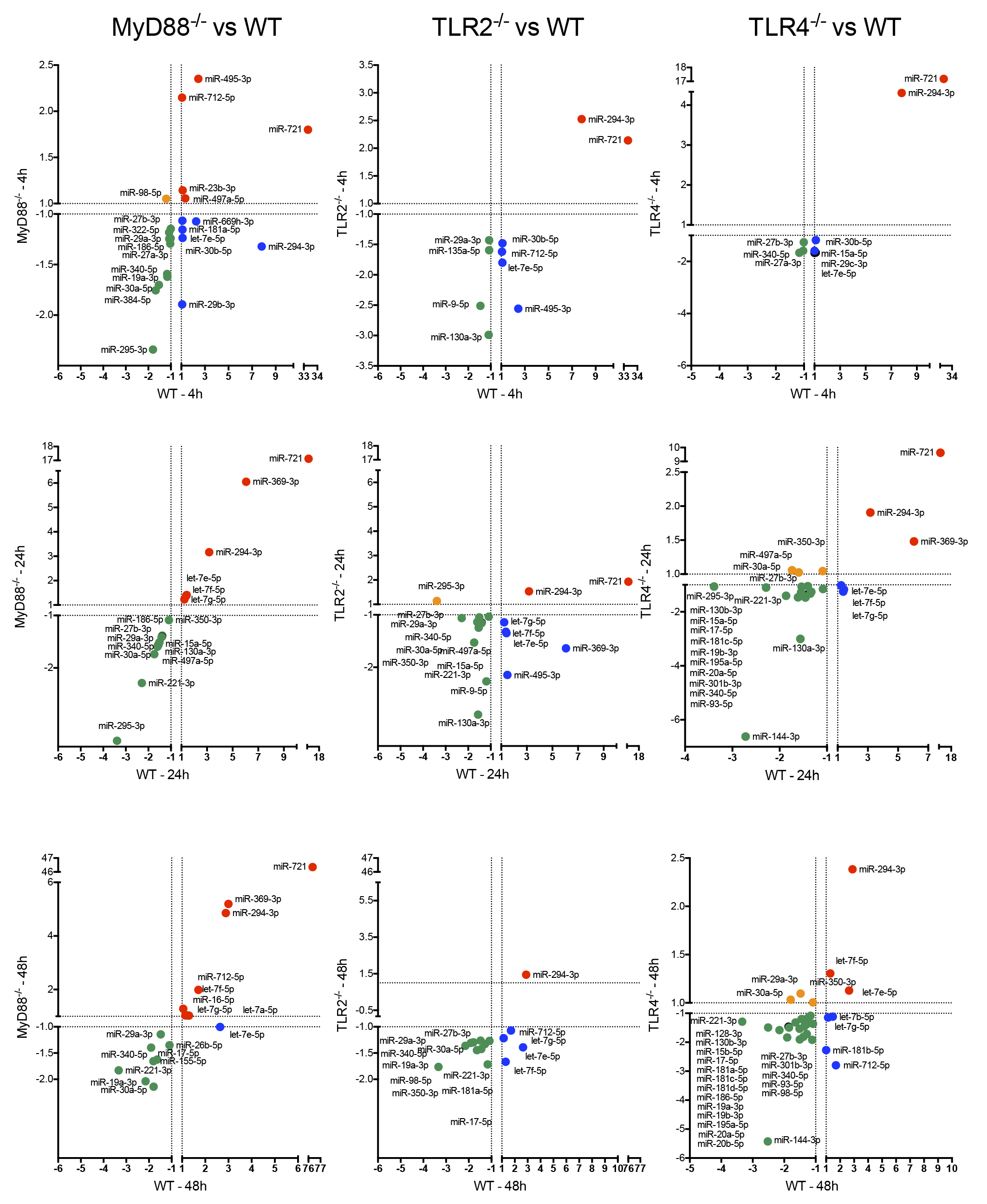

Supplement: Figure S1 — Scatter plots of the miRNA profiles of C57BL/6 (WT), MyD88−/−, TLR2−/−, and TLR4−/− murine BMDMs infected with L. amazonensis. Each dot represents one miRNA in BMDMs infected with L. amazonensis for 4, 24, and 48 h. The red dots indicate upregulated miRNAs and green dots indicate downregulated miRNAs in both comparisons. Blue dots indicate upregulated miRNAs in WT macrophages compared to knockout cells and orange dots indicate downregulated miRNAs in WT macrophages compared to knockout cells. The relative up- and downregulation of miRNAs are reported as boundaries of 1.2 or −1.2 of Fold Regulation, respectively. Only significant (p < 0.05) Fold Regulation values are presented. [file Image_1.tif]

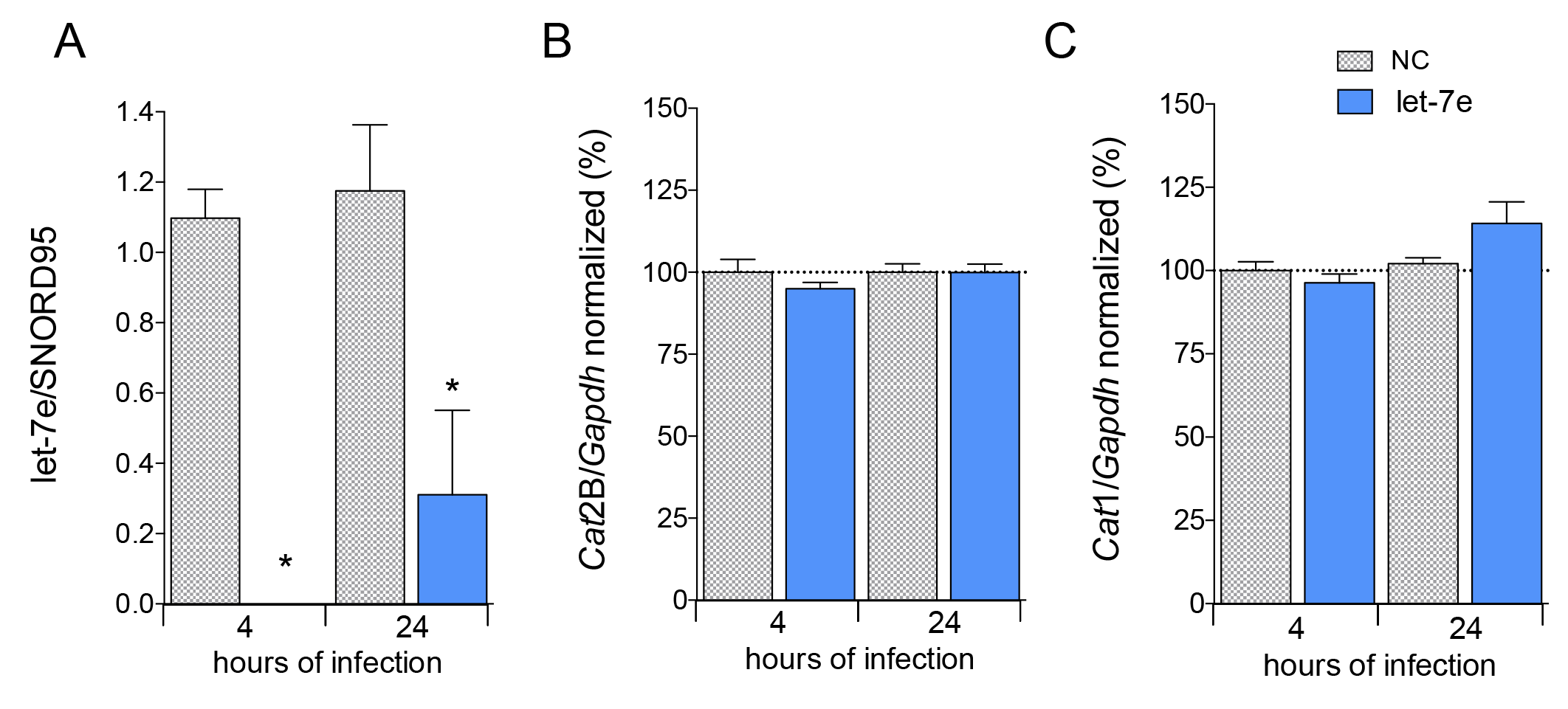

Supplement: Figure S2 — Inhibition of let-7 functions. BMDMs were transiently transfected with 100 nM negative control (NC) or let-7e-5p inhibitor. Twenty-four hours after transfection incubation, cells were co-cultivated with L. amazonensis (MOI 5:1) for 4 h, and the cultures were then washed. After 4 and 24 h of infection, the samples were analyzed for let-7e levels normalized to SNORD95 (A), and levels of the Cat2B (B), and Cat1 (C) mRNAs using RT-qPCR. The values were normalized to the average values of NC-transfected, infected macrophages (100%). Each bar represents the average ± SEM of the values obtained from three independent experiments (n = 3–6). Statistical significance was determined using two-tailed Student's t-tests. *p < 0.05 compared to negative control-transfected, infected macrophages. [file Image_2.tif]

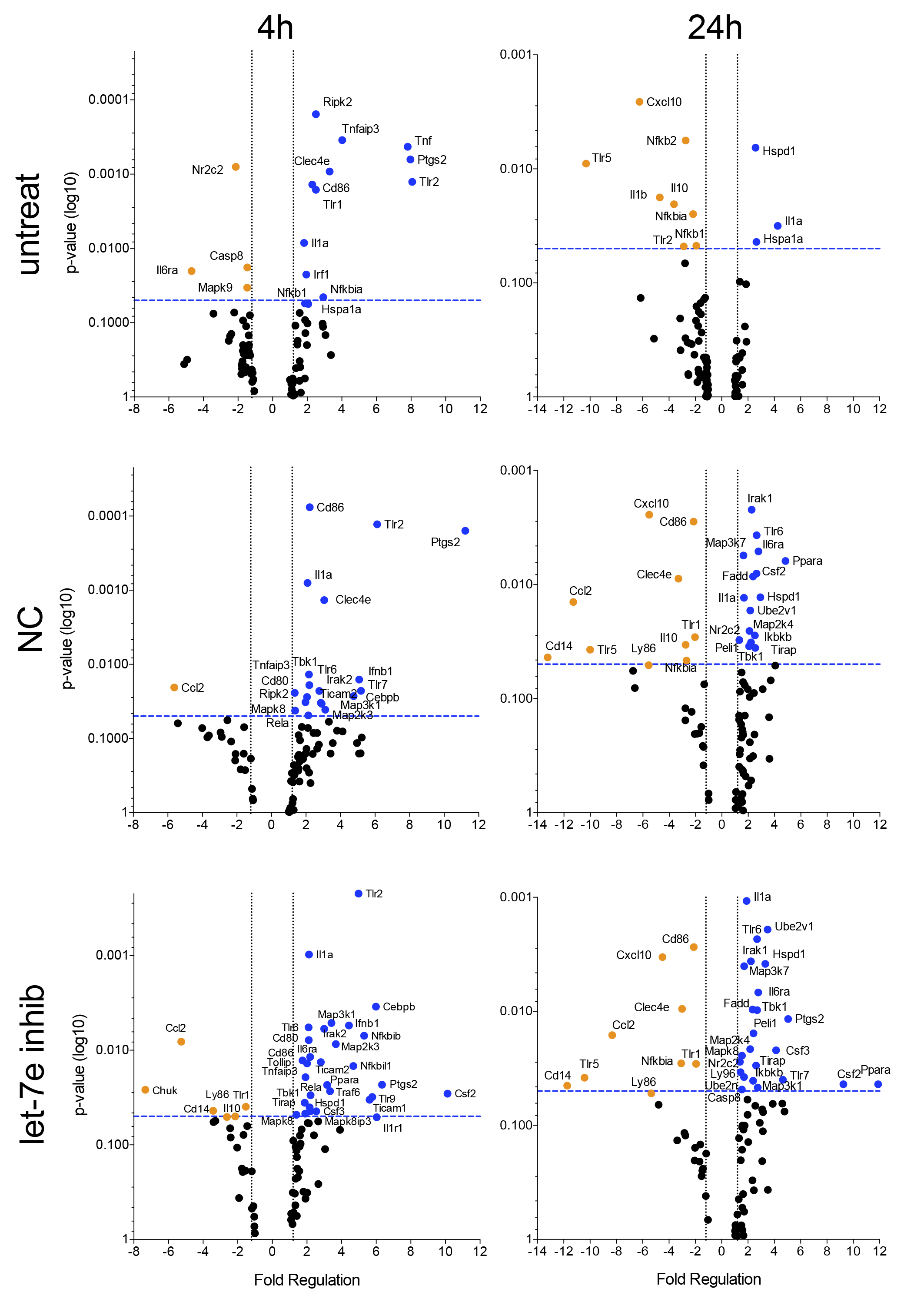

Supplement: Figure S3 — Expression of mRNAs encoding proteins in the TLR pathway molecules and cytokines in L. amazonensis-infected macrophages. The BMDMs (5 × 10^6^) from WT mice were infected with L. amazonensis (MOI 5:1). Each dot represents one mRNA in BMDMs from WT mice infected with L. amazonensis for 4 and 24 h. Blue dots indicate upregulated mRNAs and orange dots indicate downregulated mRNAs. The blue dotted line corresponds to p = 0.05, log 10. The relative up- and downregulation of miRNAs are presented as boundaries of 1.2 or −1.2 of Fold Regulation, respectively. P-values were determined using two-tailed Student's t-test. Representative data from three independent experiments are shown. [file Image_3.TIF]
